# Endoplasmic reticulum stress and NF‐kB activation in SARS‐CoV‐2 infected cells and their response to antiviral therapy

**DOI:** 10.1002/iub.2537

**Published:** 2021-08-13

**Authors:** Desirée Bartolini, Anna Maria Stabile, Carmine Vacca, Alessandra Pistilli, Mario Rende, Antimo Gioiello, Gabriele Cruciani, Francesco Galli

**Affiliations:** ^1^ Department of Chemistry, Biology and Biotechnology University of Perugia Perugia Italy; ^2^ Department of Medicine and Surgery, Section of Human, Clinical and Forensic Anatomy University of Perugia Perugia Italy; ^3^ Applied Biochemistry and Nutrition Lab, Department of Pharmaceutical Sciences University of Perugia Perugia Italy

**Keywords:** c‐Jun, COVID‐19, inflammation, Nelfinavir, NF‐kB, Remdesivir, SARS‐CoV‐2, stress response, VERO‐E6 cells

## Abstract

Unfolded protein response (UPR) and endoplasmic reticulum (ER) stress are aspects of SARS‐CoV‐2‐host cell interaction with proposed role in the cytopathic and inflammatory pathogenesis of this viral infection. The role of the NF‐kB pathway in these cellular processes remains poorly characterized. When investigated in VERO‐E6 cells, SARS‐CoV‐2 infection was found to markedly stimulate NF‐kB protein expression and activity. NF‐kB activation occurs early in the infection process (6 hpi) and it is associated with increased MAPK signaling and expression of the UPR inducer IRE‐1α. These signal transduction processes characterize the cellular stress response to the virus promoting a pro‐inflammatory environment and caspase activation in the host cell. Inhibition of viral replication by the viral protease inhibitor Nelfinavir reverts all these molecular changes also stimulating *c*‐Jun expression, a key component of the JNK/AP‐1 pathway with important role in the IRE‐1α‐mediated transcriptional regulation of stress response genes with anti‐inflammatory and cytoprotection function. The present study demonstrates that UPR signaling and its interaction with cellular MAPKs and the NF‐kB activity are important aspects of SARS‐CoV‐2‐host cell interaction that deserve further investigation to identify more efficient therapies for this viral infection.

**Abbreviations**
AP‐1: activating protein‐1
BCA: bicinchoninic acid
BSL3: biosafety level 3 laboratory
CPE: cytopathic effect
c‐Jun: Jun proto‐oncogene
ECL: enhanced chemiluminescence
ER: endoplasmic reticulum
FBS: fetal bovine serum
GAPDH: glyceraldehyde 3‐phosphate dehydrogenase
IL‐6: interleukin‐6
IL‐10: interleukin‐10
IRE‐1α: inositol‐requiring enzyme 1 alpha
JNK: c‐Jun N‐terminal kinase
MAPK: mitogen‐activated protein kinase
MOI: multiplicity of infection
Nel: Nelfinavir
NF‐kB: nuclear factor kappa‐light‐chain‐enhancer of activated B cells
PARPs: poly (ADP‐ribose) polymerases
PE: phycoerythrin
Rem: Remdesivir
SARS‐CoV‐2: Severe Acute Respiratory Syndrome CoronaVirus‐2
TCID50: median tissue culture infectious dose
TNF‐α: tumor necrosis factor alpha
TRAF2: TNF receptor associated factor 2
UPR: unfolded protein response

## 1 INTRODUCTION

Nuclear factor kappa‐light‐chain‐enhancer of activated B cells (NF‐kB) is a ubiquitous transcription factor involved in inducible gene regulation to adapt cells to environmental, mechanical, chemical, and microbiological stresses.^1^, ^2^ Viral antigens are among the cellular stimuli capable to activate NF‐kB, including those of the respiratory virus subfamily Coronavirus.^3^ A sustained NF‐κB signaling has been observed in human airway epithelial cells infected with SARS‐CoV‐2, possibly by the modified expression of regulatory proteins such as dual‐specificity phosphatases 1 and 5,^4^ and PARPs^5^ involved in apoptotic pathway modulation.^6^ Again, this transcription factor has been speculated to represent one of the therapeutic targets in this infection along with other cellular stress and pro‐inflammatory pathways.^7^

A characteristic cellular response to NF‐kB activation is the transcriptional activation of inflammatory genes.^1^, ^4^ This suggests the possibility that NF‐kB hyperactivation in SARS‐CoV‐2 infected cells may contribute to investigate cellular stress by the abnormal expression of inflammatory genes. At the same time, an increased burden of NF‐kB activation is expected to play a main role in promoting the inflammatory comorbidity of COVID‐19. The stimulation of inflammatory genes occurs early in the cellular infection process in association with changes of the cellular redox, MAPK signaling activation and endoplasmic reticulum (ER) stress.^8^ The latter are common changes induced by the coronavirus infection in cultured cells.^9^, ^10^ Also, ER stress and MAPK pathways activation represent potent stimuli for the NF‐κB signaling and inflammatory gene expression in lung epithelial cells.^11^

Therefore, it is conceivable to hypothesize that NF‐kB is a critical player of the stress response to SARS‐CoV‐2 replication in the host cell. Based on its transcriptional function and molecular interactions with the UPR/ER stress pathway, NF‐kB may induce inflammatory genes and the apoptotic signaling in the infected cell. In order to verify these assumptions, NF‐kB expression and the inflammatory response to SARS‐CoV‐2 infection were investigated in VERO‐E6 epithelial cells, a reliable and well‐characterized model of ER stress and cytopathic effect (CPE) in this type of viral infection.^12^, ^13^ The specificity of the SARS‐CoV‐2 induced response of NF‐kB pathway was verified by the treatment with pharmacological inhibitors of viral replication with proven efficacy in cytoprotection of VERO‐E6 cells^8^; these included the viral protease inhibitor Nelfinavir and the nucleotide analogue Remdesivir.

## 2 METHODS

### 2.1 Cell model and infection protocol

VERO‐E6 cells were cultured in minimal essential medium, EMEM (Gibco, Life Technologies) supplemented with 10% fetal bovine serum (FBS, Gibco, Life Technologies), 100 units/ml penicillin, 100 μg/ml streptomycin and 2 mM l‐glutamine (Lonza) at 37°C in 5% CO_2_.

SARS‐CoV‐2 strain was isolated from a nasopharyngeal swab obtained from a symptomatic patient as described in References 8, 14. Sample collection was performed according to the declaration of Helsinki and received approval from the local ethics committee. The virus was handled in a biosafety level 3 (BSL3) laboratory at the virology unit of “*Santa Maria della Misericordia*” Hospital, Perugia, Italy.

Viral titration by TCID_50_ assay was carried out in VERO‐E6 cells seeded on a 96‐well tissue culture plate on day before the assay. Confluent VERO‐E6 cells were washed once and overlaid with MEM medium supplemented with 100 units/ml penicillin, 100 μg/ml streptomycin and 1% FBS. Virus supernatants were serially diluted. Diluted supernatants were added onto VERO‐E6 plates in triplicate. Viral titer was determined by Median Tissue Culture Infectious Dose (TCID50) endpoint dilution and stock aliquots were stored at −80°C. The stock virus titer was 3.16 × 10^7^ TCID50/ml and the frozen aliquots were thawed immediately before each experiment.

### 2.2 Chemicals

Remdesivir (Rem; MedChem Express, NJ, HY‐104077) and Nelfinavir (Nel; AMBH2D6EF522, Sigma‐Aldrich, St. Louis, MO) were dissolved in dimethyl sulfoxide (DMSO, Sigma‐Aldrich, St. Louis, MO) and aliquots of stock solutions (1000×) were maintained at −80°C until further use. During cellular treatments, the compounds were diluted at the indicated concentrations in culture medium to obtain final DMSO concentrations <0.001%. Cytotoxicity and cell viability during treatments were assessed using MTT assay as described in Reference 8.

### 2.3 Evaluation of virus‐induced cytopathology

One day prior to the experiment, 20,000 cells/well were seeded in 96‐well flat‐bottom plates. Cells were infected with SARS‐CoV‐2 at a multiplicity of infection (MOI) of 0.0035 in complete medium for 1 hr and then antiviral compounds were added and incubated at 37°C in a humidified incubator with 5% CO_2_. Uninfected (mock) controls were included in each plate. At 24–48–72 hr postinfection, virus‐induced cytophatology was observed by microscopy. Three independent experiments were performed, each including a technical duplicate.

### 2.4 Immunofluorescence assay

After infection and/or treatment, VERO‐E6 cells in 96 well plates flat bottom black polystyrene wells (with micro‐clear bottom, Greiner CELLSTAR®) were fixed with 10% neutral formalin (Leica), and then washed in three times with PBS (Euroclone) and permeabilized with Triton X‐100 0.5% in PBS for 5 min at room temperature (RT). Subsequently, blocking was carried out with 3% FBS for 15 min at RT under slow stirring. At the end of the blocking, a washing was carried out with distilled water and then the incubation with the primary antibody was carried out in 3% FBS for 1 hr at RT and under slow stirring. Then, three washes were carried out in distilled water and the labeled secondary antibodies diluted 1:1,000 and the DAPI (Life Technologies) to stain the nuclei (1:3,000) were placed in 3% FBS for 1 hr in the dark. Afterward, four washes were carried out in distilled water and were added phalloidin‐Alexa Fluor 488 (Life Technologies) to stain actin filaments (1:1,000 in PBS) for 40 min in the dark under stirring. Finally, six washes were carried out in sterile distilled water (Euroclone) and the black plates (Greiner CELLSTAR®) were ready for quantitative fluorescence analysis (from 63–72 fields/well) and image acquisition with 40× and/or 63× objectives with water system by Operetta CLS system (PerkinElmer). The primary antibodies used were: NF‐kB Polyclonal Antibody, PE‐anti‐human IL‐6 Antibody (BioLegend, 1:1,000); PE anti‐human IL‐10 Antibody (BioLegend, 1:1,000). The secondary antibody used was: Alexa Fluor‐555 anti‐mouse IgG (Invitrogen).

### 2.5 Immunoblotting

VERO‐E6 cells were prepared for immunoblot analysis as described in Reference 15. Proteins were quantified using the bicinchoninic acid (BCA) assay using bovine serum albumin as an external standard. Proteins (20 μg) were loaded onto 4–12% SDS–polyacrylamide gel electrophoresis (SDS–PAGE) minigels (Invitrogen), and immobilized on nitrocellulose membrane for immunoblot analysis. The membranes were then incubated with 5% skim milk in Tris‐buffered saline (TBS; 20 mM Tris base, 150 mM NaCl, pH 7.4) and 0.1% Tween‐20 for 2 hr at RT. The blots were incubated with primary antibodies at 4°C overnight, with constant shaking and then washed twice with TBS. The primary antibodies were: anti‐NFKB (p65; #6956; 1:1,000), anti‐phospho‐NFKB (p105, #4806;1:1,000), anti‐cleaved caspase 9 (#9505; 1:1,000), anti‐*c*‐Jun (#2315; 1:1,000), anti‐phospho‐ERK1/2 (#4377; 1:1000), anti‐ERK1/2(#4695; 1:1,000), anti‐IRE1‐α (#3294; 1:1000),anti‐TNF‐α (#6945; 1:1,000), anti‐phospho‐p38 MAPK (Thr180/Tyr182) (#4511; 1:1,000), anti β‐actin (#3700; 1:1,000), anti‐GAPDH (#5174; 1:1,000) from Cell Signaling Technology. The secondary antibodies were anti‐rabbit (#7074) or anti‐mouse (#7076) IgG (1:2,000) horseradish peroxidase‐linked (Cell Signaling Technology). Protein bands were detected using an ECL Clarity Max or ECL Clarity (BioRad). Quantification of bands was performed with a Gel‐Pro Analyzer; protein expression level was normalized to housekeeping protein expression.

### 2.6 Statistics

Statistical significance estimated using One‐Way ANOVA followed by Tukey's test for multiple comparison or Student's *t*‐test (*p <.05; **p <.01). Statistical analysis was performed using GraphPad Prism (v.6.0).

## 3 RESULTS AND DISCUSSION

### 3.1 Cytopathic effect

**FIGURE 1:**
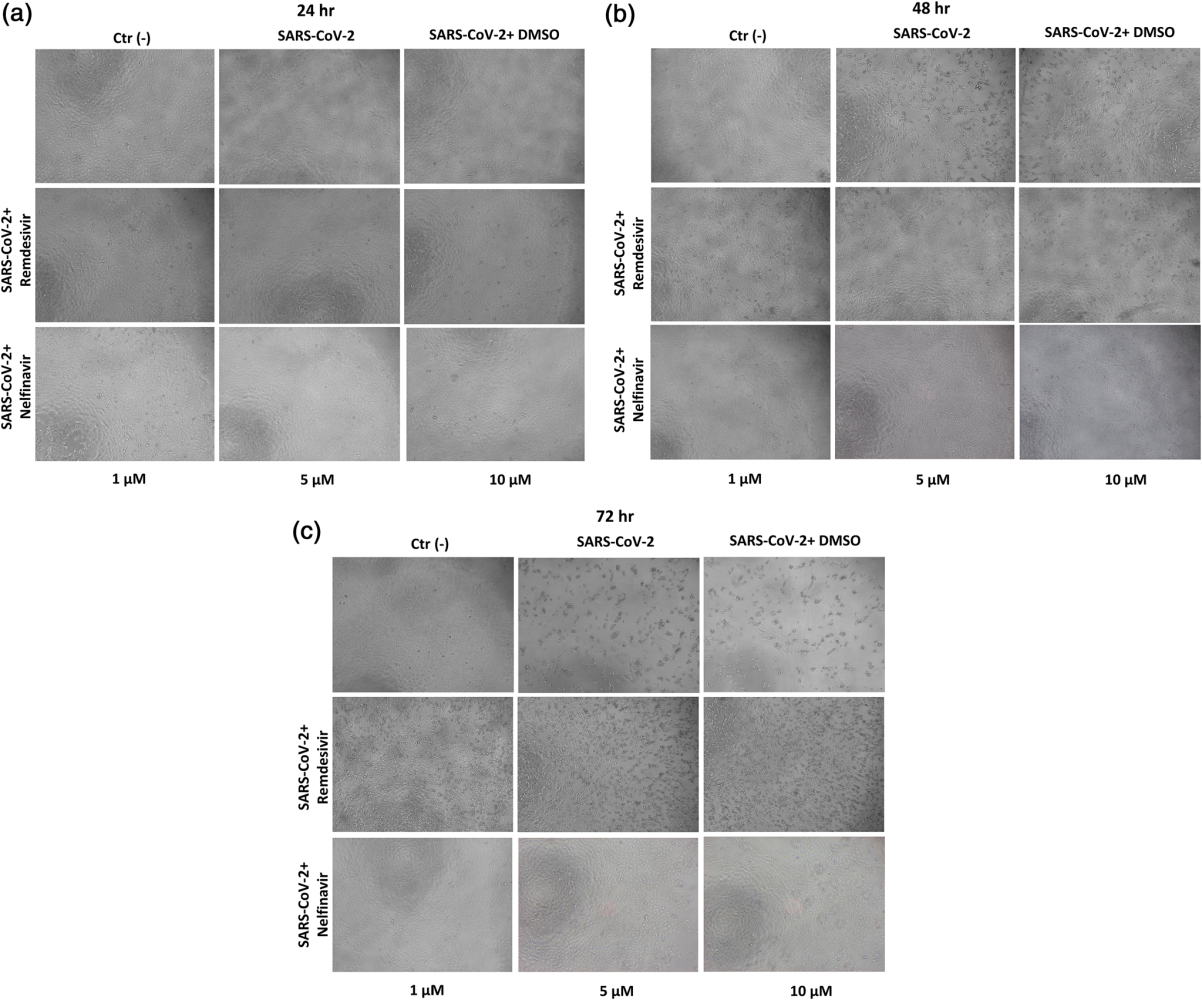
Effect of the antivirals Nelfinavir and Remdesivir on the SARS‐CoV‐2‐induced cytopathic effect (CPE) of VERO‐E6 cells. Changes in cell density and morphology were investigated 24 (a), 48 (b), and 72 (c) hpi as cues of CPE. VERO‐E6 cells were infected with SARS‐CoV‐2 at MOI: 0.0035 and were treated with different concentrations of antivirals (1–10 μM) 2 hpi

The CPE of SARS‐CoV‐2 infected cells was investigated assessing the characteristic changes of cellular morphology (Figure 1). An overt CPE in this cell line manifests from 24 hpi in association with changes on molecular and metabolic indicators of cellular damage and with increased viral RNA copy number (described in Reference 8 and references therein). According with previous drug screening data carried out in this^8^ and other laboratories,^16^, ^17^, ^18^, ^19^ Nelfinavir was a potent inhibitor of SARS‐CoV‐2 replication and CPE in VERO‐E6 cells (Figure 1). Nelfinavir was originally developed as a drug for HIV therapy, but its unavailable for clinical therapy in that its production has been interrupted.

Much less efficient, compared with Nelfinavir was the in vitro antiviral activity of Remdesivir (Figure 1), a nucleotide analog approved by the FDA and European Medicines Agency (EMA) for the treatment of adult and pediatric COVID‐19 with reported efficacy in reducing the time to recovery in hospitalized patients.^20^

### 3.2 NF‐kB protein expression and activity

**FIGURE 2:**
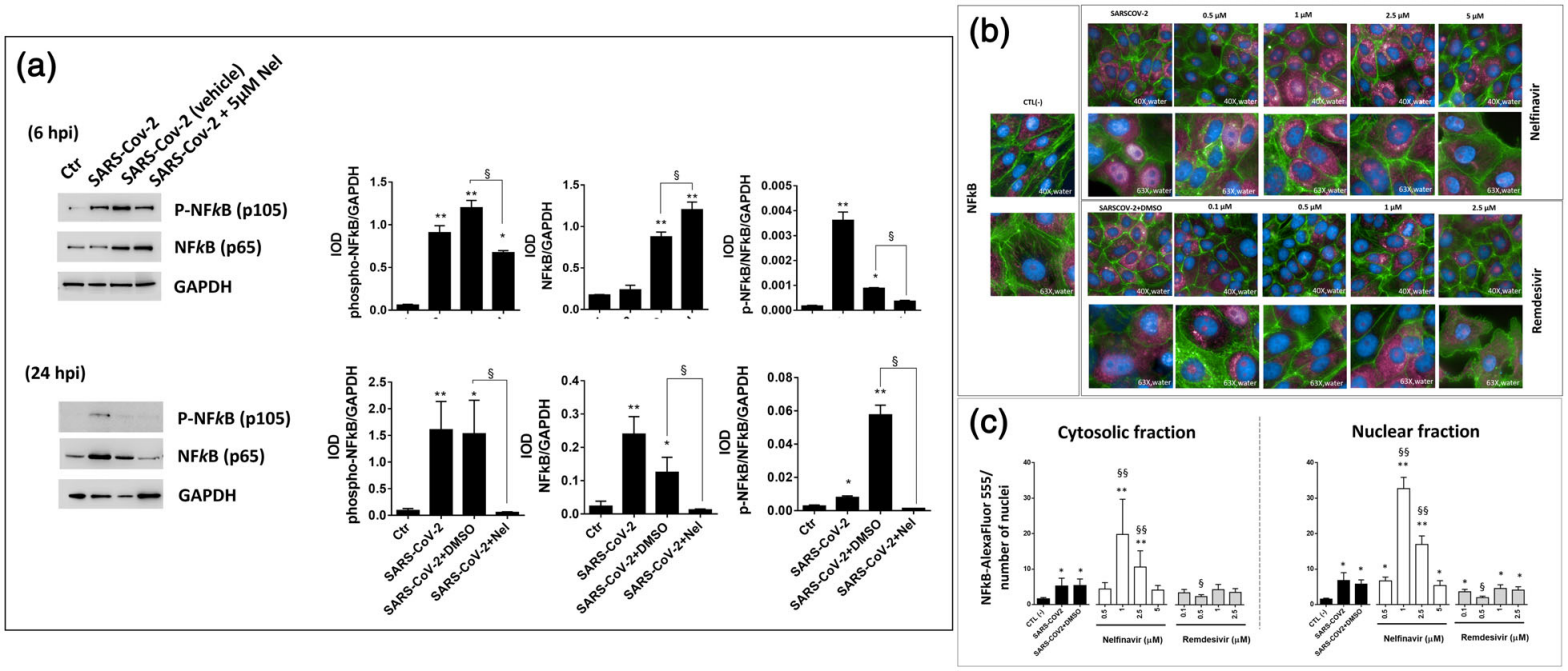
NF‐kB activation in SARS‐CoV‐2 infected VERO‐E6 cells. (a) Immunoblot of total cell proteins carried out 6 hpi (upper panels) and 24 hpi (lower panels). (b) Immunofluorescence of NF‐kB‐Alexa Fluor 555 was assessed 48 hpi. DAPI (blue) was used to highlight the nuclei while the Phalloidin‐Alexa Fluor488 (green) was used to stain the cytoplasm. (c) quantification of cytoplasmic and nuclear NF‐kB‐Alexa Fluor 555 (fuchsia) staining; bars are mean ± standard deviation of data quantified from 63 field images collected in three independent experiments. The cells were treated with Nelfinavir (or Remdesivir, only in panel b and c) after infection with SARS‐CoV‐2 at MOI 0.0035. CTL versus all treatments: *p <.05, **p <.001; SARS‐CoV‐2 versus SARS‐CoV‐2 + treatment: §*p* <.05, §§*p* <.001

Since the earliest phase of the infection process (i.e., from 6 hpi onward), SARS‐CoV‐2 markedly stimulates NF‐kB protein expression and phosphorylation (Figure 2a). Immunofluorescence experiments (Figure 2b,c) showed increased NF‐kB protein expression and nuclear translocation 48 hpi, thus indicating persistent activation of this transcription factor throughout the whole virus replication cycle and CPE process.

**FIGURE 3:**
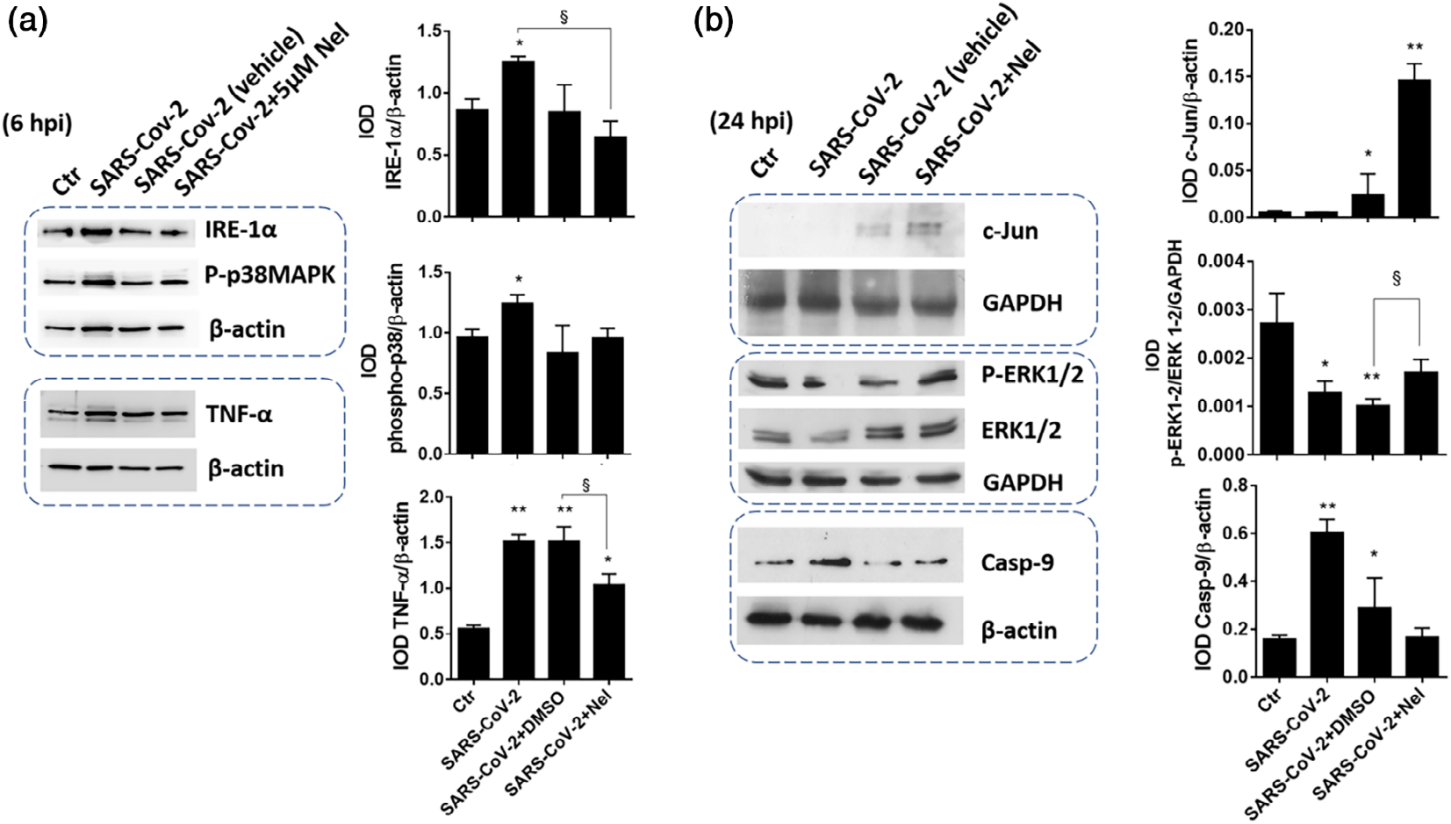
Signal transduction data of SARS‐CoV‐2 infected VERO‐E6 cells. The cells were exposed to SARS‐CoV‐2 as described in Figure 1 and the protein extract was collected for immunoblot analysis 24 hpi. Cellular epitopes investigated included: (a) the UPR indicator IRE‐1α, the phosphorylated form of p38‐MAPK, and TNF‐α; (b) the transcription factor c‐Jun, MAPK‐ERK1/2 and the apoptotic effector caspase‐9. CTL versus all treatments *p <.05; **p <.001; SARS‐CoV‐2 + DMSO versus SARS‐CoV‐2 + Nel §*p* <.05

According with a transcriptional activation of NF‐kB, the infected cells showed increased levels of the pro‐inflammatory cytokines TNF‐α (Figure 3a) and IL‐6 (Table 1), whereas the levels of IL‐10 decreased (Table 1). The latter is an important anti‐inflammatory cytokine with key role in infection by limiting the immune response to pathogens and thereby preventing damage to the host.^21^

**TABLE 1:** Levels of interleukin 6 and 10 in SARS‐CoV2 infected VERO‐E6 cells and effect of Nelfinavir and Remdesivir treatment

	IL‐6 (fluorescence intensity/nuclei number)	IL‐10 (fluorescence intensity/nuclei number)
Ctr	0.27 ± 0.03	0.87 ± 0.15
SARS‐CoV‐2	0.32 ± 0.04^*^	0.68 ± 0.03^*^
SARS‐CoV‐2 + DMSO	0.32 ± 0.05^*^	0.55 ± 0.11^*^
SARS‐CoV‐2 + Nel 1 μM	0.31 ± 0.04^*^	0.58 ± 0.11^*^
SARS‐CoV‐2 + Nel 5 μM	0.17 ± 0.06^**^	0.79 ± 0.05^**^
SARS‐CoV‐2 + Rem 1 μM	0.36 ± 0.03^*^	0.67 ± 0.14^*^
SARS‐CoV‐2 + Rem 2.5 μM	0.45 ± 0.04^*^	0.66 ± 0.15^*^

^*^ *p* < .05 versus Ctr experiment; ^**^ *p* < .01 versus SARS‐CoV‐2+DMSO.

IL‐6 and IL‐10 levels of infected cells were restored after treatment with Nelfinavir, but not with Remdesivir (Table 1). This response to Nelfinavir treatment was concentration‐dependent, with 5 μM as the effective concentration, and was associated with reduced expression of TNF‐α protein (Figure 3a), and reduced expression and phosphorylation of NF‐kB protein (Figure 2a).

Moreover, Nelfinavir, but not Remdesivir, showed a concentration‐dependent U‐shaped stimulation effect on NF‐kB expression and nuclear translocation 48 hpi (Figure 2b,c), suggesting the involvement of NF‐kB signaling in the antiviral activity and cell homeostasis restoration mechanism of this viral protease inhibitor.

### 3.3 IRE‐1α/UPR pathway, MAPK signaling, and cleaved caspase 9 expression

SARS‐CoV‐2 infection in VERO‐E6 cells stimulated the expression of the ER stress signaling protein IRE‐1α. The stress MAPK‐p38 phosphorylation, TNF‐α expression (Figure 3a), and the apoptotic effector caspase‐9 (Figure 3b) were also upregulated upon infection. At the same time, both the expression and phosphorylation of the survival MAPK‐ERK1/2 were reduced (Figure 3a). These findings confirm the central role of IRE‐1α activation in UPR initiation and downstream modulation of a wide variety of signaling pathways, such as stress and survival MAPKs, apoptosis, and innate immune response. The integrated response of IRE‐1α/UPR pathway with other stress response and apoptosis induction mechanisms, is described for the first time in SARS‐CoV‐2 infection. This type of interaction has already been described in cultured cells infected with other coronaviruses (reviewed in Reference 9) and pathogens.^22^ As an original finding in this study, IRE‐1α activation in SARS‐CoV‐2 infected cells is associated with NF‐kB activation and pro‐inflammatory cytokine induction, which are key pathogenic events in COVID‐19, a viral disease that can evolve to severe inflammatory complications and oxidative stress especially in the elderly (recently reviewed in Reference 23). IRE‐1α induction and dimerization on the ER membrane stimulates NF‐kB activity by TRAF2 kinase activation and consequent IKK‐mediated phosphorylation and proteolytic removal of the NF‐kB inhibitor IkBα.^22^, ^24^ Furthermore, IRE‐1α activation during pathogen‐induced ER stress may induce the expression of inflammatory cytokines, such as TNF‐α (Figure 3a) and IL‐6 (Table 1), via XBP1 mRNA splicing modulation,^22^ which may represent a mechanism alternative to NF‐kB activation to promote inflammation in the SARS‐CoV‐2 infected cell.

The involvement of NF‐kB in the UPR and ER stress response of the host cell was further confirmed upon inhibition of viral replication with Nelfinavir. The treatment with this antiviral significantly reduced IRE‐1α activity of the infected cell (Figure 3a) also restoring p38 and MAPK‐ERK1/2 signaling, TNF‐α expression and caspase 9 levels (Figure 3ab).

Worth of note is that Nelfinavir promotes IL‐10 induction (presented in Section 2 and in Table 1), which is important to restore inflammatory pathways and the cellular homeostasis after interruption of the viral replication process.^21^ IL‐10 induction involves MAPK‐ERK1/2 and p38, and NF‐κB signaling and transcriptional activation via promoter binding of the same NF‐κB and the AP‐1 transcriptional complex^21^; the latter include *c*‐Jun, a transcriptional protein downstream of stress‐activated MAPK‐JNK and IRE‐1α/TRAF2 pathway of UPR important for the pathogen‐induced stress response of the host cell.^22^ Nelfinavir treatment was found to stimulate *c*‐Jun protein expression (Figure 3b), restoring at the same time all signal transduction and transcriptional elements of IL‐10 pathway of the infected cell, including NF‐kB (Figure 2a), and the stress and survival MAPKs p38 and ERK1/2, respectively (Figure 3a,b). These findings demonstrate that the Nelfinavir‐induced inhibition of IRE‐1α pathway of UPR in SARS‐CoV‐2 infected cells involves both the JNK/*c*‐Jun (AP‐1) and NF‐kB pathways (Figure 2b,c) and their control function on cell death programs, including the caspase‐dependent apoptosis^6^ (Figure 3b).

In conclusion, UPR signaling and ER stress are main aspects of the SARS‐CoV‐2‐host interaction, apparently contributing to viral replication and to its inflammatory complications. NF‐kB transcription factor is involved in these cellular responses to the viral infection. Its activation in SARS‐CoV‐2 infected VERO‐E6 cells appears to involve the IRE‐1α/TRAF2 pathway of UPR and the signaling of cellular MAPKs. These signal transduction and transcriptional pathways promote a pro‐inflammatory environment in the host cell also leading to CPE and apoptotic cell death activation. The inhibition of viral replication by the antiviral Nelfinavir reverts these molecular and cellular changes, thus confirming their association with viral replication and its stress response of the host cell. The treatment with this antiviral also stimulated the JNK/*c*‐Jun (AP‐1) pathway; together with NF‐kB activity, AP‐1 is demonstrated to promote the transcription of important cytoprotection and anti‐apoptotic genes that are impaired after viral infection, including IL‐10 and the genes associated with the metabolism and antioxidant function of cellular glutathione that are restored in SARS‐CoV‐2 infected VERO‐E6 cells upon Nelfinavir treatment.^8^ The findings in this study further confirm the importance of restoring homeostatic processes of the host cell during the inhibition of viral replication, which should be a key prerequisite to design proper antiviral therapies in COVID‐19.

## CONFLICT OF INTEREST

The authors have no conflict of interest to declare.
